# Kisspeptin-10 increases collagen content in the myocardium by focal adhesion kinase activity

**DOI:** 10.1038/s41598-023-47224-3

**Published:** 2023-11-15

**Authors:** Paulina Radwańska, Małgorzata Gałdyszyńska, Lucyna Piera, Jacek Drobnik

**Affiliations:** Department of Pathophysiology, Institute of General and Experimental Pathology, Medical University of Lodz, Żeligowskiego 7/9, 90-752 Lodz, Poland

**Keywords:** Cell biology, Cardiology, Endocrinology

## Abstract

The aim of the study was to evaluate the role of kisspeptin-10 (KiSS-10) in the regulation of collagen content in cardiac fibroblasts. An attempt was also made to describe the mechanism of the effect of KiSS-10 on collagen metabolism. The studies indicate that kisspeptin-10 significantly increases the content of intracellular collagen in the myocardium. KiSS-10 also elevates the level of phosphorylated focal adhesion kinase (FAK) in human cardiac fibroblasts. The inhibition of FAK negates the stimulatory effect of KiSS-10 on collagen deposition in vitro. These changes correlate with an increase in the level of propeptides of procollagen type I (PICP) and III (PIIICP) in fibroblast culture medium and mouse PIIICP in serum. Moreover, this hormone inhibits the release of metalloproteinases (MMP-1,-2,-9) and elevates the secretion of their tissue inhibitors (TIMP-1,-2,-4). KiSS-10 also enhances the expression of α1 chains of procollagen type I and III in vitro. Thus, KiSS-10 is involved in the regulation of collagen metabolism and cardiac fibrosis. Augmentation of collagen deposition by KiSS-10 is dependent on the protein synthesis elevation, inhibition of MMPs activity (increase of TIMPs release) or decrease of MMPs concentration. The profibrotic activity of KiSS-10 is mediated by FAK and is not dependent on TGF-β1.

## Introduction

The heart is composed of cardiomyocytes, the cells of the electrical conduction system, as well as a network of blood vessels, macrophages, and fibroblasts. The cardiac fibroblasts are involved in the production of the extracellular matrix (ECM) components such as collagen and basement membrane proteins, as well as nonstructural molecules, such as glycosaminoglycans, glycoproteins and adhesive proteoglycans. However, fibrillar collagen type I and III are the main components of the cardiac ECM^1^. In addition, in the myocardium, fibroblasts are responsible for the production of a number of cytokines, growth factors, peptides, and enzymes such as matrix metalloproteinases (MMPs) and their tissue inhibitors (tissue inhibitor of metalloproteinases—TIMPs) that directly influence ECM turnover^2^. The ECM of the connective tissue acts as a supportive scaffold for the cells that determines the structural integrity of the heart and helps regulate its mechanical and electrical function. Also, interactions between myocardial cells and ECM mediate biomechanical communication as mechanotransduction and influence the metabolism of the heart^3^.

However, an imbalance between collagen production and degradation leads to its accumulation in the myocardium and development of cardiac fibrosis. There are three types of myocardial fibrosis: reactive interstitial fibrosis characterized by ECM deposition, infiltrative interstitial fibrosis arising as a consequence of glycolipid accumulation in different heart cells, or replacement fibrosis that develops after cardiac cells necrosis^4^. Regardless of the etiology, fibrotic processes in the heart drive the differentiation of fibroblasts into myofibroblasts, which are responsible for overproduction and deposition of collagen. Myofibroblasts are also involved in the secretion of MMPs and TIMPs that contribute to ECM remodeling and fibrogenesis. The pathological activation of myofibroblasts is triggered under the influence of profibrotic growth factors and cytokines^5^. One important mediator of cardiac fibrosis is transforming growth factor beta 1 (TGF-β1)^6^. Collagen metabolism in the heart is also influenced by reactive oxygen species and hormones^7^. It was recently reported that collagen deposition and pathological myocardial remodeling are associated with high levels of peptide hormones known as kisspeptins^8^.

Kisspeptins are a family of amidated neurohormones, identified as endogenous ligands of G-protein coupled receptors 54 (GPR54) also known as the KiSS1R, AXOR12 or hOT7T175. The precursor is a peptide comprising 145 amino acids, encoded by the *kiss-1* gene. This is, proteolytically cleaved into four biologically-active peptides differentiated by peptide chain length: kisspeptin-54, kisspeptin-14, kisspeptin-13, kisspeptin-10. All functional kisspeptins contain a 10-amino acid fragment at the C-terminus, which is biologically active at the receptor level^9, 10^. The KiSS-1/GPR54 system plays a variety of functions related to hormone secretion^11^, inhibition of cells proliferation and migration, enhancement of apoptosis^12^, improvement of memory capabilities^13^ or regulation of insulin secretion^14^. However, less is known about the influence of kisspeptin on cardiovascular system, and only a few reports indicate that kisspeptins alter cardiovascular function, both centrally and peripherally. Maguire et al.^15^ showed that KiSS-1/GPR54 system is expressed in the myocardium and vasculature of humans, mice and rats. In these species, kisspeptin exerts a positive inotropic action in paced atrial strips in vitro. Shojaei et al*.*^16^ noted that kisspeptin deficiency can be a risk factor of acute myocardial infarction (AMI). They revealed that kisspeptin serum levels in AMI patients were significantly lower compared to the healthy individuals. Mead et al.^17^ indicated that kisspeptin acts as a potent endothelium-independent vasoconstrictor in large human vessels with the same developmental origin: viz*.* the umbilical vein and coronary artery. Kisspeptin has also been found to have vasoactive properties at the microvascular level. Sawyer et al.^18^ noted that intradermal injection of kisspeptin decreases cutaneous peripheral blood flow and induces plasma extravasation in mice. Moreover, Zhang et al.^8^ demonstrate on rats that the pathological remodeling of the heart is connected with high level of kisspeptin. This hormone could alter the morphology and structure of myocardial cells, serum metabolite levels and expression of genes and proteins in heart tissue. Rats receiving an injection with kisspeptin had increased numbers of collagen fibers in the cardiac muscle^8^.

The detection of expression of KiSS-1/GPR54 system within the myocardium suggests that this hormone can affect heart functions. However, available data is scarce and do not explain the mechanism of the effect of kisspeptin on connective tissue metabolism within the heart. Therefore, the aim of the present study was to determine the role of kisspeptin-10 (KiSS-10) in the regulation of collagen content in cardiac fibroblasts. An attempt was also made to describe the mechanism of the effect of KiSS-10 on collagen metabolism.

## Materials and methods

### Reagent preparations

The following reagents were used in the in vitro experiment: Kisspeptin-10 (synthetic peptide; sequence: Tyr–Asn–Trp–Asn–Ser–Phe–Gly–Leu–Arg–Phe–NH2) (catalog no. orb372481, Biorbyt, Cambridge, UK) and FAK inhibitor 14 (FAKi) (1,2,4,5-Benzenetetramine tetrahydrochloride, Tocris, Ellisville, MO, USA). Both kisspeptin-10 and FAKi were initially dissolved in water and stored at − 20 °C until further dilution in medium immediately prior to use. KiSS-10 was prepared at a concentrations of 1.0 × 10^−11^–1.0 × 10^−5^ mol/L. FAKi was used at concentration 10^−6^ mol/L. KiSS-10 (Kisspeptin-10 (110–119), Metastin, mouse, rat, 1 mg) for experiment *iv vivo* was obtained from Eurogentec (Seraing, Belgium). This reagent was dissolved in water for injection and used at concentrations of 1 nmol/200 µL and 10 nmol/200 µL.

### Cell culture

The presented study was conducted on an immortalized human cardiac fibroblast cell line (ABM, Richmond, BC, Canada). The cells were obtained from ventricles of human heart of healthy adult. The cells were cultured in Dulbecco’s Modified Eagle’s Medium (DMEM) (Biowest, Nuaillé, France) supplemented with 10% (v/v) foetal bovine serum (FBS) (Biowest, Nuaillé, France), gentamicin (25 µg/ml) (Gibco, Thermo Fisher Scientific, Waltham, Massachusetts, USA), amphotericin B (0.25 µg/ml) (Capricorn Scientific, Ebsdorfergrund, Germany), insulin (5 µg/ml) (Thermo Fisher Scientific, Waltham, Massachusetts, USA), and vitamin C (50 µg/ml) (Sigma-Aldrich, Saint Louis, Missouri, USA) on the culture plates coated with collagen (10 µg/cm^2^) (Sigma-Aldrich, Saint Louis, Missouri, USA) at 37 °C under the humidified atmosphere of 5% CO_2_. The cells were grown to confluence before being trypsynized (Trypsin–EDTA 1X in PBS, Biowest, Nuaillé, France) and passaged. All experiments were carried out on the cells from passage 7 to 13. The human cardiac fibroblasts were incubated in DMEM containing 3% (v/v) bovine serum, insulin, vitamin C, antibiotics in the concentrations given above and 10^−11^-10^−5^ mol/L of kisspeptin-10 or FAK inhibitor 14 (FAKi, 10^−6^ mol/L) or KiSS-10 (10^−8^ mol/L) with FAKi (10^−6^ mol/L) for 96 h. The results were compared with control cultures without hormones or inhibitors. The medium was changed every day. On the day 5 of the experiment, the total number of fibroblasts and the number of necrotic (stained with trypan blue) cells were counted in the Burker chamber. Intracellular collagen content, expression of α1 chains of procollagen type I (*Col1A1*) and III (*Col3A1*) and level of phosphorylated focal adhesion kinase (phospho-FAK) were measured in the cultures on the last day of the experiment. Before determination of the collagen level, the cells were trypsynized and washed four times to remove extracellular collagen that was used to coat the plates. Also, the culture media were collected on day 5 to analyse the concentrations of propeptides of procollagen type I and III (PICP, PIIICP), MMPs, TIMPs, TGF-β1.

### Animals and experimental design

Experimental male BALB/c mice (12 weeks old, n = 32) were obtained from the Animal House of the University of Lodz, Poland. All animals were maintained in a standard housing condition i.e. in groups of 2–5 animals, at constant temperature (20–24 °C) and humidity (45–65%), with 12-h light/12-h dark cycle. They were kept with free access to the standard diet and tap water. Before beginning the experiment, the mice were adapted to new environment for two weeks. The animals were then divided into four groups: 1) control (n = 8); 2) placebo—group receiving subcutaneous injections of *aqua pro injectione* (200 µL, for four weeks, 1 × day) (n = 8); 3) experimental group receiving subcutaneous injections of KiSS-10 (1 nmol/200 µL, for four weeks, 1 × day) (n = 8); 4) experimental group receiving subcutaneous injections of KiSS-10 (10 nmol/200 µL, for four weeks, 1 × day) (n = 8). This animal model was chosen as preferred for cardiovascular research. Kisspeptin-10 doses were selected on the basis of available data^8, 18^. The number of animals in each group was determined according to statistical verification.

The animals were anesthetized with xylazine (10 mg/kg) (Sedazin, Biowet Pulawy, Pulawy, Poland) and ketamine (100 mg/kg) (Biowet Pulawy, Pulawy, Poland) through intraperitoneal route. Under general anesthesia, blood samples were collected by cardiac puncture, and then mice were euthanized by cervical dislocation. Serum samples and hearts were collected and stored at − 80 °C until further analysis.

### Determination of the total collagen level

The collagen content in the cells cultured in 24-well culture plates and the heart samples were measured by Woessner method^19^. The fibroblast cultures were dried in a laboratory oven, while the obtained hearts were minced into pieces, dried in a laboratory oven and homogenized with a mortar. The prepared samples were taken for further analysis. The level of total collagen in samples was assessed based on the amount of hydroxyproline, which was hydrolyzed for 24 h at 100 °C in 6 N HCl (1.2–1.5 mL/culture and 3 mL/10 mg of dry tissue, respectively) (Stanlab, Lublin, Poland). All the hydrolizates were neutralized by adding 5 N NaOH (Stanlab, Lublin, Poland). The products of neutralization were diluted to 5 mL with redistilled water. Following this, 0.2 mL samples were collected and diluted with redistilled water to 1 mL final volume. The oxidation of hydroxyproline to pyrrole was carried out by adding 0.5 mL chloramine T (Chempur, Piekary Slaskie, Poland) and methyl glycol (EMSURE®, Merck, Darmstadt, Germany) in a citrate buffer (pH = 6). Then, the samples were shaken and incubated for 20 min at room temperature. Also, excess chloramine T was removed by adding 0.5 mL of 3.15 mol/L perchloric acid (Loba Chemie, Mumbai, India). After 5 min, the samples were incubated with 0.5 mL of 20% (w/v) p-dimethylaminobenzaldehyde (Sigma-Aldrich, Saint Louis, Missouri, USA) in a 60 °C water bath for 20 min. The optical density was measured by spectrophotometer at a wavelength of 560 nm.

### Enzyme-linked immunoassay (ELISA)

The concentrations of PICP, PIIICP, TGF-β1, MMP-1, MMP-2, MMP-9, TIMP-1, TIMP-2, TIMP-3, TIMP-4 in culture media were measured using commercially-available ELISA kits (catalog no. E-EL-H0196, E-EL-H0841, E-EL-H0110, E-EL-H1441, E-EL-H1445, E-EL-H1451, E-EL-H0184, E-EL-H1453, E-EL-H1454, E-EL-H1455, Elabscience, Wuhan, China) (sensitivity: 37.5 pg/mL, 7.5 pg/mL, 18.75 pg/mL, 0.1 ng/mL, 0.47 ng/mL, 18.75 pg/mL, 0.1 ng/mL, 0.1 ng/mL, 37.5 pg/mL and 46.88 pg/mL, respectively; detection range: 62.5–4000 pg/mL, 12.5–800 pg/mL, 31.25–2000 pg/mL, 0.16–10 ng/mL, 0.78–50 ng/mL, 31.25–2000 pg/mL, 0.16–10 ng/mL, 0.16–10 ng/mL, 62.5–4000 pg/mL and 78.13–5000 pg/mL, respectively; species specificity: human, no cross-reaction with analogues; these kits were validated with human serum or plasma samples by the manufacturer). Their secretion was expressed as the concentrations released into the culture medium by a certain number of cells. The concentration of phosphorylated FAK (phospho-FAK) was estimated using RayBio® Human Phospho-FAK (Tyr397) ELISA Kit (catalog no. PEL-FAK-Y397, RayBiotech, Peachtree Corners, Georgia, USA, species specificity: human; the validation studies were done using Jurkat cells). Also, serum PICP and PIIICP concentrations were analysed by ELISA kits using species-specific antibodies (catalog no. QY-E21761, QY-E21762, Qayee-Bio, Shanghai, China) (detection range: 7.8–500 pg/mL and 31.2–200 ng/mL, respectively; species specificity: mouse, no cross-reaction with analogues). The ELISA assays were carried out according to manufacturer’s instructions. Absorbance was determined using Epoch™ Microplate Spectrophotometer (BioTek Instruments Inc, Winooski, Vermont, USA).

### Quantitative polymerase chain reaction (qPCR)

Total RNA was extracted from fibroblast cell culture and from the mouse hearts using Total RNA Mini kit (A&A Biotechnology, Gdynia, Poland) according to the manufacturer’s protocol. RNA purity and quantity was detected on a NanoDrop Spectrophotometer (Thermo Fisher Scientific, Waltham, Massachusetts, USA). Then, cDNA was obtained through reverse transcription using the a PrimeScript™ RT reagent Kit (Perfect Real Time) (Takara Bio Inc., Kusatsu, Shiga, Japan) according to the manufacturer’s protocol. Samples of cDNA were amplified using Universal Probe Library (UPL) (Roche, Indianapolis, USA) and RealTime Ready Custom Single Assay (Roche, Indianapolis, USA) based on TaqMan probes (for *Col1A1, Col3A1, GAPDH, RPLP0, YWHAZ, Rpl13a, Hprt1*). The qPCR reaction was carried out using FastStart Essential Probe Master (Roche, Indianapolis, USA) according to the manufacturer’s instructions. After activation of DNA polymerase and denaturation of the cDNA for 10 min at 95 °C, 55 cycles of PCR reaction were performed: 95 °C for 10 s, 60 °C for 30 s, 72 °C for 1 s. Then, the reaction mixture was cooled at 40 °C for 30 s. Human ribosomal protein 0 (*RPLP0*), human tyrosine 3-monooxygenase/tryptophan 5-monooxygenase activation protein zeta (*YWHAZ*) and human glyceraldehyde-3-phosphate dehydrogenase (*GAPDH*) were used as a reference gene for analysis of human gene expression of α1 chains of procollagen type I (*Col1A1*) and III (*Col3A1*) in human cardiac fibroblasts. Mouse glyceraldehyde-3-phosphate dehydrogenase (*Gapdh*), mouse ribosomal protein L13a (*Rpl13a*) and mouse hypoxanthine phosphoribosyltransferase 1 (*Hprt1*) were used as a reference gene for analysis of mouse gene expression of *Col1A1* and *Col3A1* in the mouse hearts. Human *RPLP0* was measured using the primers (forward primer: 5′-GGCACCATTGAAATCCTGAG-3′, reverse primer: 5′-GAAGGGGGAGATGTTGAGC-3′) with the UPL (Universal Probe Library) probe #36 (Roche, Indianapolis, IN, USA), while human YWHAZ was measured using the primers (forward primer: 5′-AAGTGCAATGGAGACCTTGG-3′, reverse primer: 5′-GTTGCCCTAGATGCAGAAGG-3′) with the UPL probe #2 (Roche, Indianapolis, IN, USA). Human *GAPDH*, mouse *Gapdh*, mouse *Rpl13a* and mouse *Hprt1* expression was determined using Real-Time ready Custom Single Assay (Roche, Indianapolis, IN, USA)^20^. Each reaction was conducted in duplicate. The relative gene expression with multiple reference genes was determined and the geometric mean of the concentration ratio was calculated by LightCycler® 96 software (Roche, Indianapolis, USA).

### Statistical analysis

The obtained results were calculated using StatSoft Statistica 13.1 PL and expressed as mean and standard deviation (x ± SD). The normality of the data distribution was determined using the Shapiro–Wilk test and the homogeneity of variances using Levene’s test. Then, comparisons between two groups were assessed by the paired t-test or Mann–Whitney U-test for nonparametric data. Multiple comparisons were performed using the parametric one-way ANOVA with Tukey’s post hoc test, or the nonparametric Kruskal–Wallis for non-normally distributed data. Differences were considered as significant at *p* < 0.05.

### Ethical approval

The protocol of the study was approved by the Local Ethics Committee for Animal Testing in Lodz (Licence No. 25/ŁB138/2019). All methods were carried out in accordance with relevant guidelines and regulations. The study is reported in accordance with ARRIVE guidelines.

## Results

### The influence of kisspeptin-10 on the intracellular collagen content and the expression of *Col1A1 *and *Col3A1* in human cardiac fibroblasts

**Figure 1:**
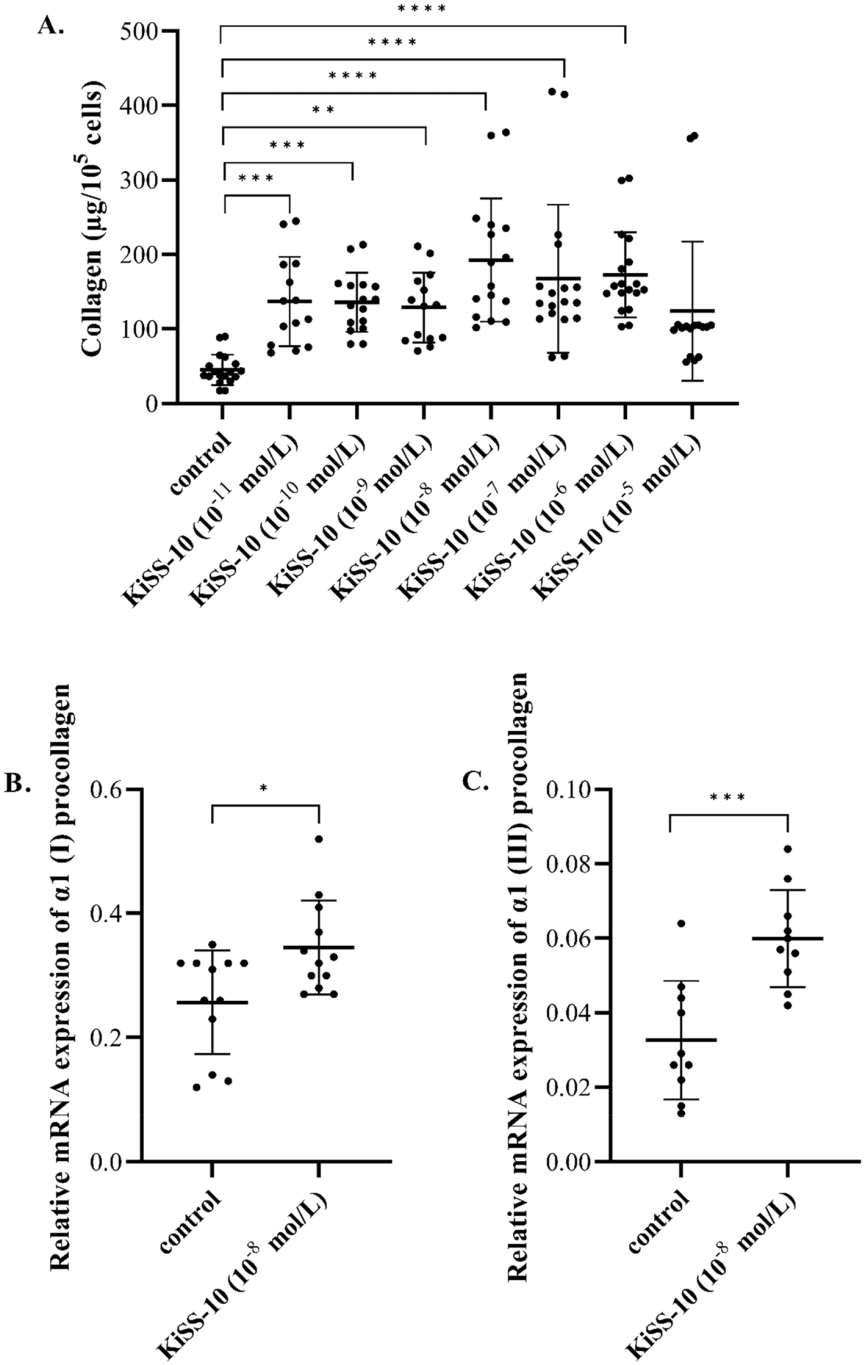
The influence of KiSS-10 (10^−11^–10^−5^ mol/L) on intracellular collagen content in human cardiac fibroblasts (**A**). Experiments were performed in biological replicates (n = 7–9). Each biological replicate was assayed in duplicate. Error bars represent standard deviation. Data were analysed by the Kruskal–Wallis test (*p < 0.05, **p < 0.01, ***p < 0.001, ****p < 0.0001—significant difference compared to the control). Changes in the expression of α1 chains of procollagen type I (*Col1A1*) (**B**) and III (*Col3A1*) (**C**) in human cardiac fibroblasts under the influence of KiSS-10 (10^−8^ mol/L) and in the control condition. Experiments were performed in biological replicates (*Col1A1:* n = 6, *Col3A1:* n = 5). Each biological replicate was assayed in duplicate. Error bars represent standard deviation. Data were analysed by the Mann–Whitney U test (*Col1A1*) and t-test (*Col3A1*) (*p < 0.05, **p < 0.01, ***p < 0.001—significant difference compared to the control).

The effect of kisspeptin-10 on intracellular collagen content was found to be dependent on the dose of KiSS-10 used. Treatment of the cells with 10^−11^ to 10^−6^ mol/L of KiSS-10 caused a significant increase in the collagen content compared to the control. The most stimulating effect (*p* < 0.0001) was observed under the influence of 10^−8^ mol/L of kisspeptin-10 (192.39 ± 82.73 vs. 45.24 ± 20.42 µg/10^5^ cells). However, the highest concentration of KiSS-10 (10^−5^ mol/L) did not change markedly the collagen content in human cardiac fibroblast in vitro (Fig. 1A).

In addition, exposure of the cells to 10^−8^ mol/L of KiSS-10 significantly elevated the expression of *Col1A1* (0.35 ± 0.08 vs. 0.26 ± 0.08; p < 0.05) as well as *Col3A1* (0.06 ± 0.01 vs. 0.03 ± 0.02; *p* < 0.001) in human cardiac fibroblasts in vitro (Fig. 1B,C).

### The effect of kisspeptin-10 on the secretion of markers of collagen synthesis in vitro

**Figure 2:**
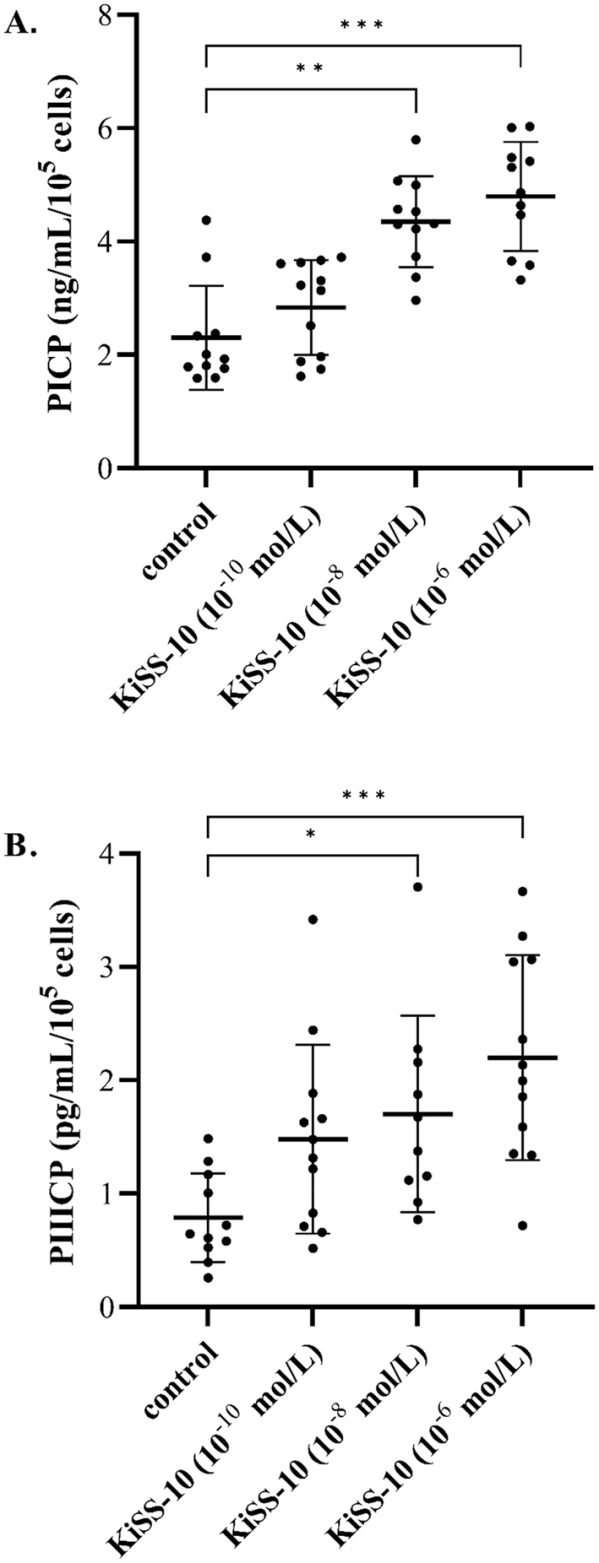
The content of C-terminal propeptides of procollagen type I (PICP) (**A**) and III (PIIICP) (**B**) within human cardiac fibroblasts media under the influence of KiSS-10 (10^−10^, 10^−8^, 10^−6^ mol/L) and in the control condition. Experiments were performed in biological replicates (PICP: n = 11–12, PIIICP: n = 10–12). Error bars represent standard deviation. Data were analysed by the Kruskal–Wallis test (PICP) and one-way ANOVA with Tukey’s post hoc test (PIIICP) (*p < 0.05, **p < 0.01, ***p < 0.001—significant difference compared to the control).

Exposure of human cardiac fibroblasts to the 10^−8^ and 10^−6^ mol/L of KiSS-10 significantly (*p* < 0.01 and *p* < 0.001, respectively) increased the content of PICP in the culture media compared to the control (4.35 ± 0.80 vs. 2.30 ± 0.91 ng/mL/10^5^ cells and 4.80 ± 0.96 vs. 2.30 ± 0.91 ng/mL/10^5^ cells, respectively) (Fig. 2A). Similarly, 10^−8^ and 10^−6^ mol/L of KiSS-10 demonstrated a significant (*p* < 0.05 and *p* < 0.001, respectively) stimulatory effect on the secretion of the PIIICP from the cells (1.70 ± 0.87 vs. 0.79 ± 0.39 pg/mL/10^5^ cells and 2.20 ± 0.90 vs. 0.79 ± 0.39 pg/mL/10^5^ cells, respectively) (Fig. 2B). However, there was no significant changes in the release of PICP and PIIICP at the lowest concentration of KiSS-10 (10^−10^ mol/L) (Fig. 2).

### The secretion of MMPs and TIMPs from human cardiac fibroblasts under the influence of kisspeptin-10

**Figure 3:**
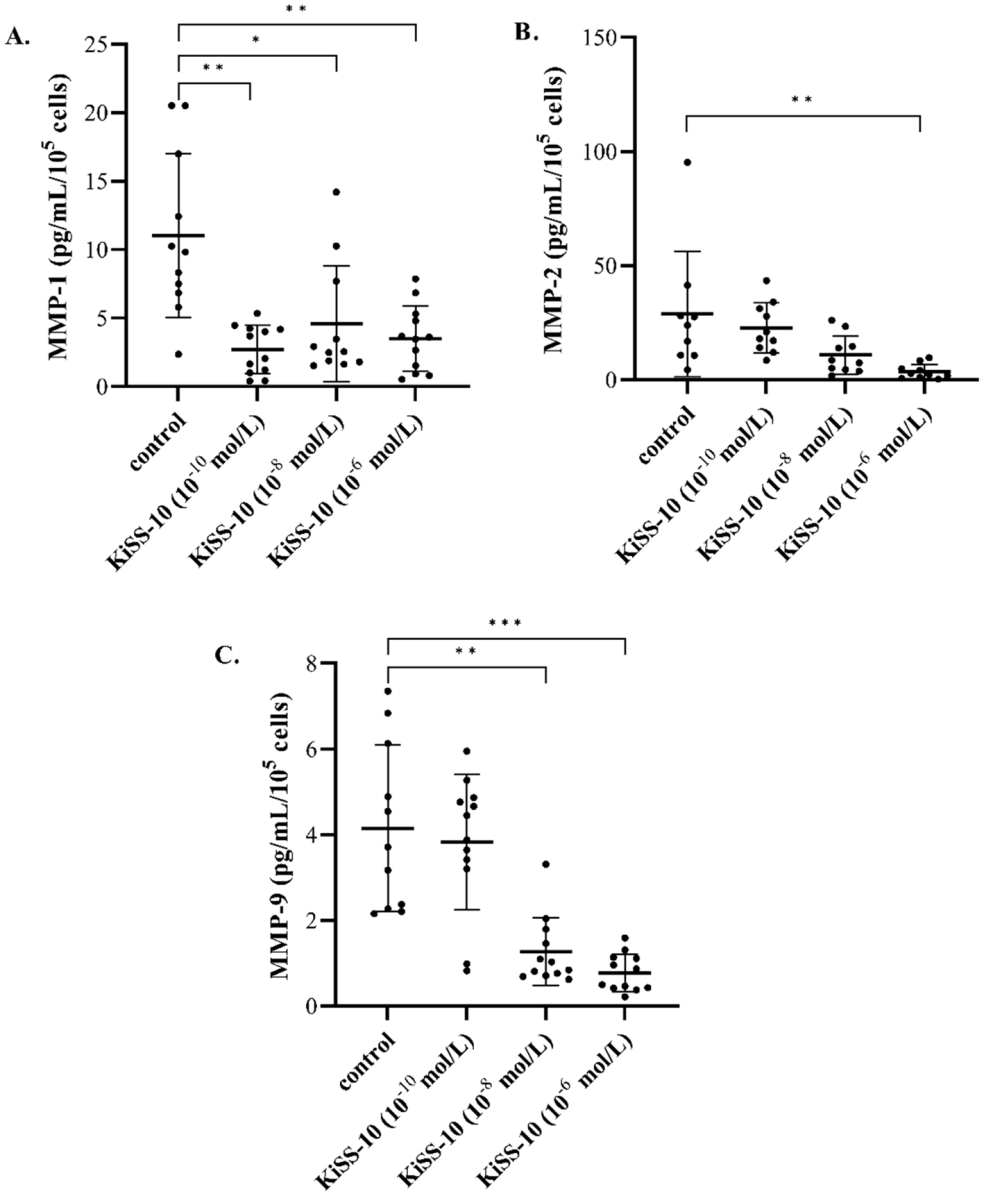
The effect of KiSS-10 (10^−10^, 10^−8^, 10^−6^ mol/L) on the secretion of metalloproteinases: MMP-1 (**A**), MMP-2 (**B**) and MMP-9 (**C**) from human cardiac fibroblasts. Experiments were performed in biological replicates (MMP-1: n = 11–12, MMP-2: n = 9–10, MMP-9: n = 11–12). Error bars represent standard deviation. Data were analysed by the Kruskal–Wallis test (*p < 0.05; **p < 0.01; ***p < 0.001—significant difference compared to the control).

The addition of kisspeptin-10 to the culture media resulted in a significant reduction of metalloproteinase secretion (MMP-1, MMP-2, MMP-9) and increase in the release of their tissue inhibitors (TIMP-1, TIMP-2, TIMP-4) from human cardiac fibroblasts. Kisspeptin-10 in at 10^−10^ mol/L, 10^−8^ mol/L and 10^−6^ mol/L significantly decreased (*p* < 0.01, *p* < 0.05 and *p* < 0.01, respectively) the content of MMP-1 within the culture media compared to the control (2.72 ± 1.77 vs. 11.03 ± 5.99 pg/mL/10^5^ cells, 4.59 ± 4.24 vs. 11.03 ± 5.99 pg/mL/10^5^ cells and 3.50 ± 2.38 vs. 11.03 ± 5.99 pg/mL/10^5^ cells, respectively) (Fig. 3A). A significant (*p* < 0.01) decrease of MMP-2 secretion was observed only under the influence of 10^−6^ mol/L of KiSS-10 (3.60 ± 3.23 vs. 28.79 ± 27.37 pg/mL/10^5^ cells). However, lower concentrations of kisspeptin-10 did not impact markedly on MMP-2 content within the cardiac fibroblast media (Fig. 3B). KiSS-10 also reduced the secretion of MMP-9 from human cardiac fibroblasts in vitro*.* The concentration of MMP-9 was significantly (*p* < 0.01 and *p* < 0.001, respectively) lower in cultures treated with 10^−8^ mol/L and 10^−6^ mol/L than in non-treated controls (1.27 ± 0.80 vs. 4.15 ± 1.94 pg/mL/10^5^ cells and 0.78 ± 0.44 vs. 4.15 ± 1.94 pg/mL/10^5^ cells, respectively) (Fig. 3C).

**Figure 4:**
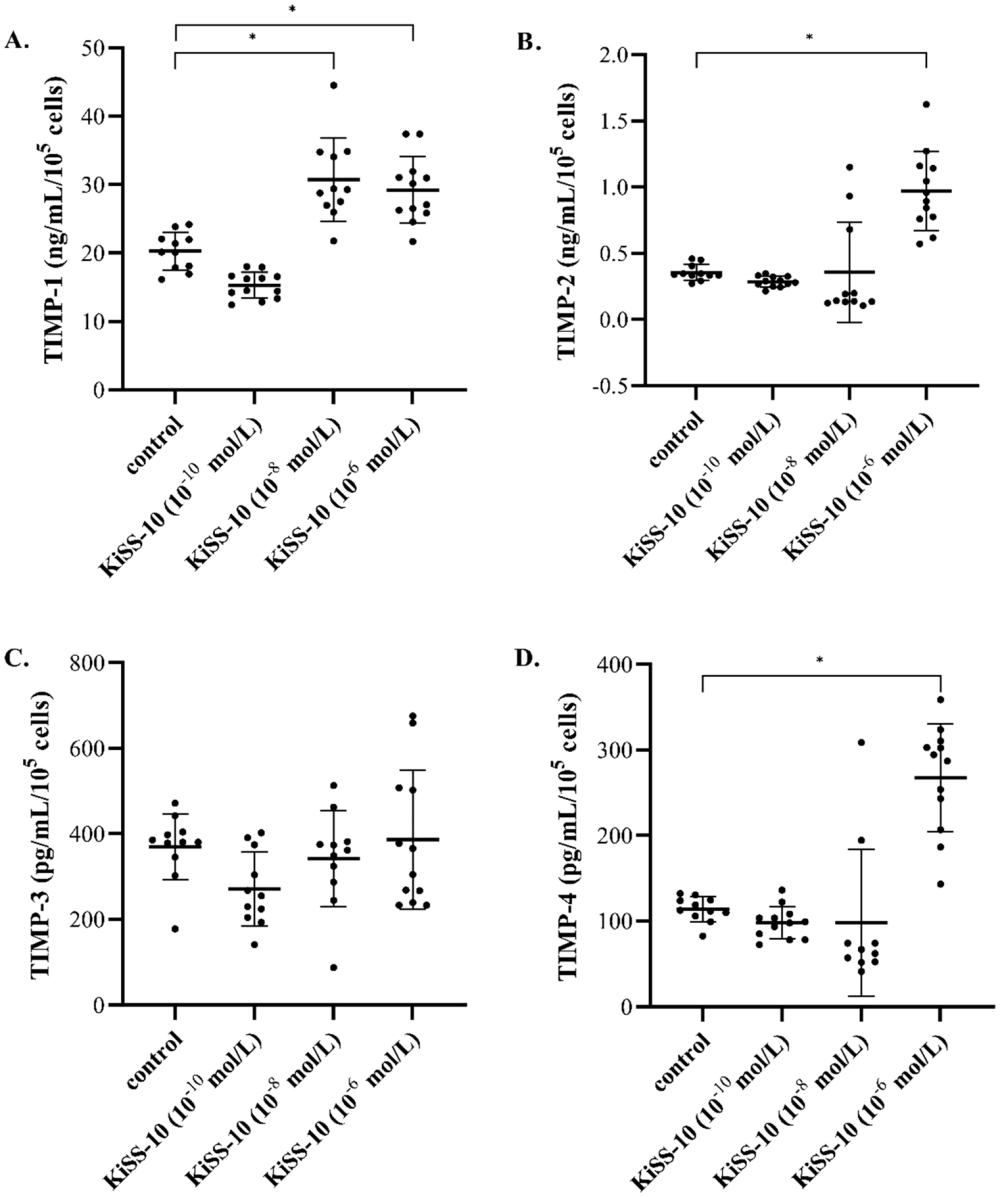
The influence of KiSS-10 (10^−10^, 10^−8^, 10^−6^ mol/L) on the release of tissue inhibitors of metalloproteinases: TIMP-1 (**A**), TIMP-2 (**B**), TIMP-3 (**C**) and TIMP-4 (**D**) from human cardiac fibroblasts. Experiments were performed in biological replicates (TIMP-1: n = 11–12, TIMP-2: n = 11–12, TIMP-3: n = 11–12, TIMP-4: n = 10–12). Error bars represent standard deviation. Data were analysed by the Kruskal–Wallis test (*p < 0.05—significant difference compared to the control).

The influence of kisspeptin-10 on TIMP release from human cardiac fibroblasts was dependent on the dose of KiSS-10 used. Treatment with 10^−8^ and 10^−6^ mol/L of kisspeptin-10 resulted in a significant (all *p* < 0.05) increase of TIMP-1 secretion compared to the control (30.73 ± 6.08 vs. 20.25 ± 2.73 ng/mL/10^5^ cells and 29.24 ± 4.85 vs. 20.25 ± 2.73 ng/mL/10^5^ cells, respectively). In addition, the lowest concentration of KiSS-10 did not significantly alter the level of TIMP-1 within the human cardiac fibroblasts media (Fig. 4A). Moreover, it was revealed that 10^−6^ mol/L of KiSS-10 markedly (all *p* < 0.05) enhanced the secretion of TIMP-2 and TIMP-4 (0.97 ± 0.30 vs. 0.35 ± 0.06 ng/mL/10^5^ cells and 267.82 ± 62.83 vs. 113.78 ± 14.64 pg/mL/10^5^ cells, respectively) (Fig. 4B,D). However, no significant differences in TIMP-2 and TIMP-4 release were found between cultures treated with 10^−10^ mol/L and 10^−8^ mol/L of KiSS-10 and non-treated controls. Moreover, the addition of KiSS-10 in all concentrations (10^−10^ mol/L, 10^−8^ mol/L, 10^−6^ mol/L) did not affect the content of TIMP-3 within the human cardiac fibroblasts compared to controls (Fig. 4C).

### The impact of KiSS-10 on the release of TGF-β1 in vitro

**Figure 5:**
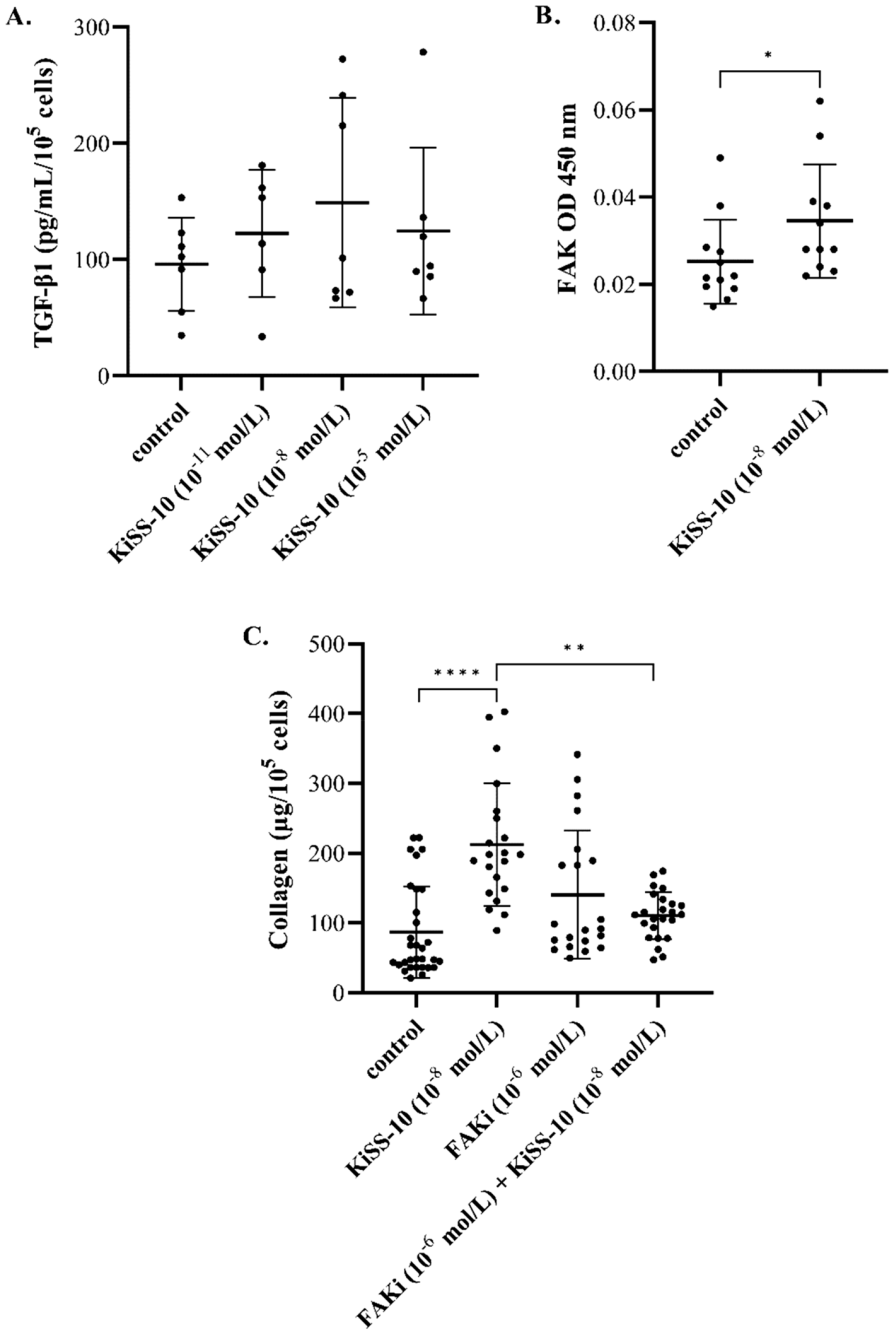
Content of transforming growth factor β1 (TGF-β1) within human cardiac fibroblast media under the influence of KiSS-10 (10^−11^, 10^−8^, 10^−5^ mol/L) and in the control condition (**A**). Experiments were performed in biological replicates (n = 6–7). Error bars represent standard deviation. Data were analysed by the Kruskal–Wallis test. The level of phosphorylated focal adhesion kinase (FAK) in human cardiac fibroblasts under the influence of KiSS-10 (10^−8^ mol/L) and in the control condition (**B**). Experiments were performed in biological replicates (n = 11–12). Error bars represent standard deviation. Data were analysed by the Mann-Whitney U test (*p < 0.05—significant difference compared to the control). The intracellular collagen content in human cardiac fibroblasts under the influence of KiSS-10 (10^−8^ mol/L), FAK inhibitor 14 (FAKi 10^−6^ mol/L) or KiSS-10 (10^−8^ mol/L) with FAKi (10^−6^ mol/L) (**C**). Experiments were performed in biological replicates (n = 11–12). Each biological replicate was assayed in duplicate. Error bars represent standard deviation. Data were analysed by the Kruskal-Wallis test (*p < 0.05, **p < 0.01, ***p < 0.001, ****p < 0.0001—significant difference between the groups).

No significant changes in the secretion of TGF-β1 were observed from human cardiac fibroblasts under the influence of 10^−11^ mol/L, 10^−8^ mol/L or 10^−5^ mol/L KiSS-10 compared to controls (Fig. 5A).

### The effect of KiSS-10 on the level of phospho-FAK (Tyr397) in vitro

Exposure of the cells to 10^−8^ mol/L KiSS-10 significantly changed the level of phospho-FAK in human cardiac fibroblasts. Cells treated with 10^−8^ mol/L of KiSS-10 demonstrated a significant (*p* < 0.05) increase in phospho-FAK (0.035 ± 0.013) compared to controls (0.025 ± 0.010) (Fig. 5B).

### Changes in the intracellular collagen content in human cardiac fibroblasts under the influence of KiSS-10 and KiSS-10 with FAK inhibitor 14

Human cardiac fibroblasts treated with 10^−8^ mol/L of KiSS-10 demonstrated significantly (*p* < 0.0001) higher content of intracellular collagen (212.36 ± 87.81 µg/10^5^ cells) compared to untreated cells (87.78 ± 66.75 µg/10^5^ cells). In contrast, the addition of KiSS-10 with FAKi (10^−6^ mol/L) resulted in a significant (*p* < 0.01) reduction of collagen level compared to the culture with KiSS-10 alone (110.65 ± 33.85 vs. 212.36 ± 87.81 µg/10^5^ cells). No changes in the collagen content in human cardiac fibroblasts under the influence of FAKi (10^−6^ mol/L) were found compared to the control (Fig. 5C).

### The effect of kisspeptin-10 on the collagen content and the expression of *Col1A1 *and *Col3A1* within the hearts of mice

**Figure 6:**
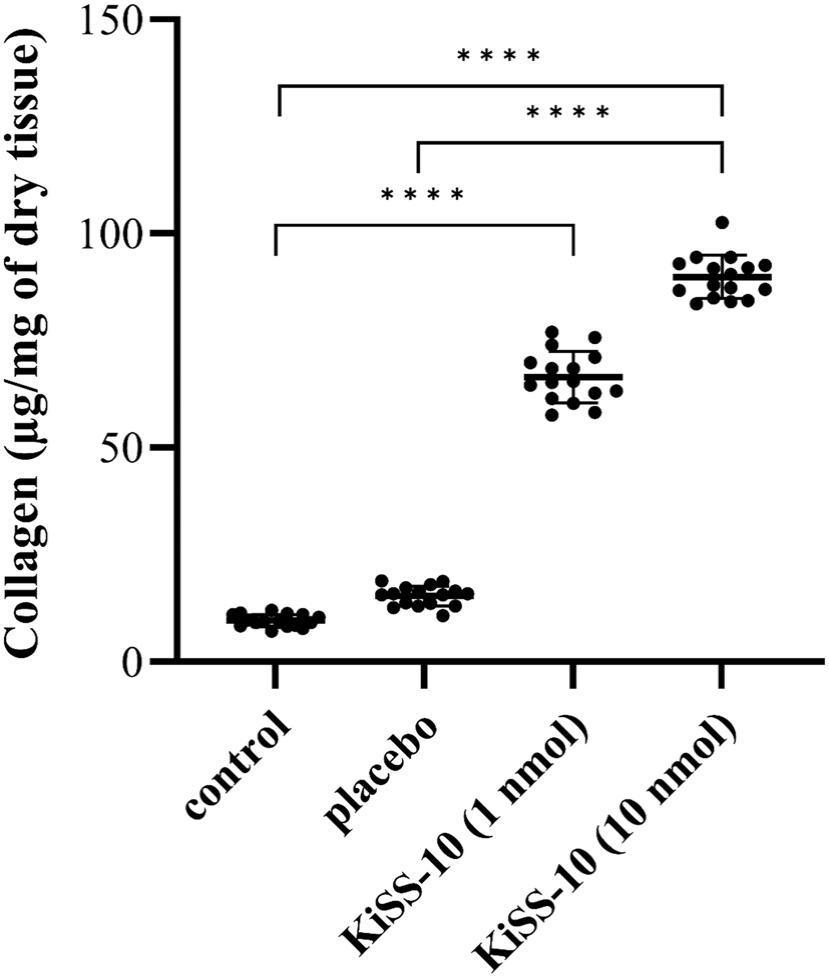
Content of collagen within the hearts of mice: control group; placebo (group receiving injections of *aqua pro injectione*); experimental group receiving injections of KiSS-10 (1 nmol/200 µL); experimental group receiving injections of KiSS-10 (10 nmol/200 µL). Experiments were performed in biological replicates (n = 8). Each biological replicate was assayed in duplicate. Error bars represent standard deviation. Data were analysed by the Kruskal–Wallis test (*p < 0.05, **p < 0.01, ***p < 0.001, ****p < 0.0001—significant difference between the groups).

The animal model studies revealed that kisspeptin-10 elevated the collagen content within the hearts of mice in vivo. Administration of 10 nmol/200 µL kisspeptin-10 to the mice caused a significant (*p* < 0.0001) increase in the collagen content in the hearts (89.82 ± 5.07 µg/mg) compared to the control (9.66 ± 1.44 µg/mg) and placebo (15.39 ± 2.35 µg/mg). Mice treated with a lower dose of KiSS-10 (1 nmol/200 µL) demonstrated significantly (*p* < 0.0001) higher collagen levels within the hearts compared to controls (66.42 ± 5.95 vs. 9.66 ± 1.44 µg/mg) (Fig. 6).

**Figure 7:**
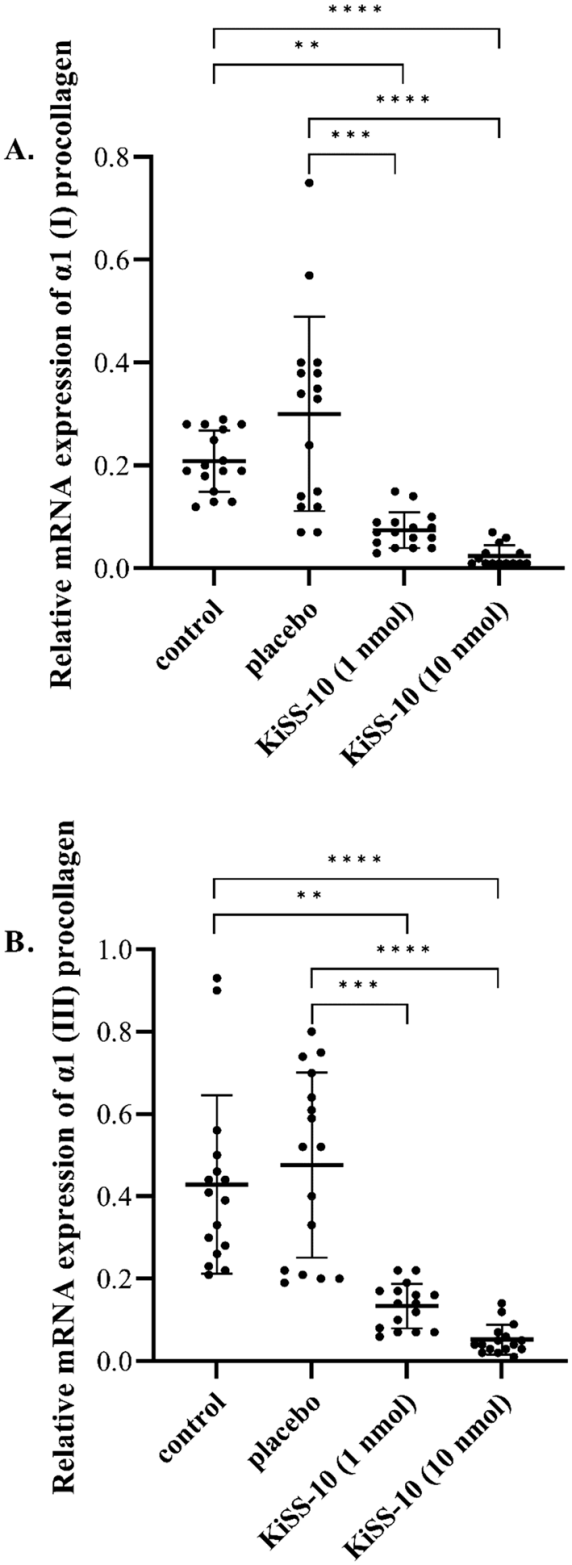
Changes in the expression of α1 chains of procollagen type I (*Col1A1*) (**A**) and III (*Col3A1*) (**B**) within the hearts of mice: control group; placebo (group receiving injections of *aqua pro injectione*); experimental group receiving injections of KiSS-10 (1 nmol/200 µL); experimental group receiving injections of KiSS-10 (10 nmol/200 µL). Experiments were performed in biological replicates (*Col1A1*: n = 7–8, *Col3A1*: n = 8). Each biological replicate was assayed in duplicate. Error bars represent standard deviation. Data were analysed by the Kruskal–Wallis test (*p < 0.05, **p < 0.01, ***p < 0.001, ****p < 0.0001—significant difference between the groups).

At the same time the mice treated with KiSS-10 in a dose 1 nmol/200 µL and 10 nmol/200 µL had a significant decrease in the expression of *Col1A1* (0.07 ± 0.03 and 0.02 ± 0.02, respectively) compared to the control (0.21 ± 0.06) and placebo (0.30 ± 0.19) (Fig. 7A). A similar effect was observed with expression of *Col3A1* within the hearts of mice. Administration of KiSS-10 in a dose 1 nmol/200 µL and 10 nmol/200 µL to the mice reduced the expression of *Col3A1* (0.13 ± 0.05 and 0.05 ± 0.04, respectively) compared to the control group (0.43 ± 0.22) and placebo (0.48 ± 0.23) (Fig. 7B).

### Changes in serum concentration of PICP and PIIICP

**Figure 8:**
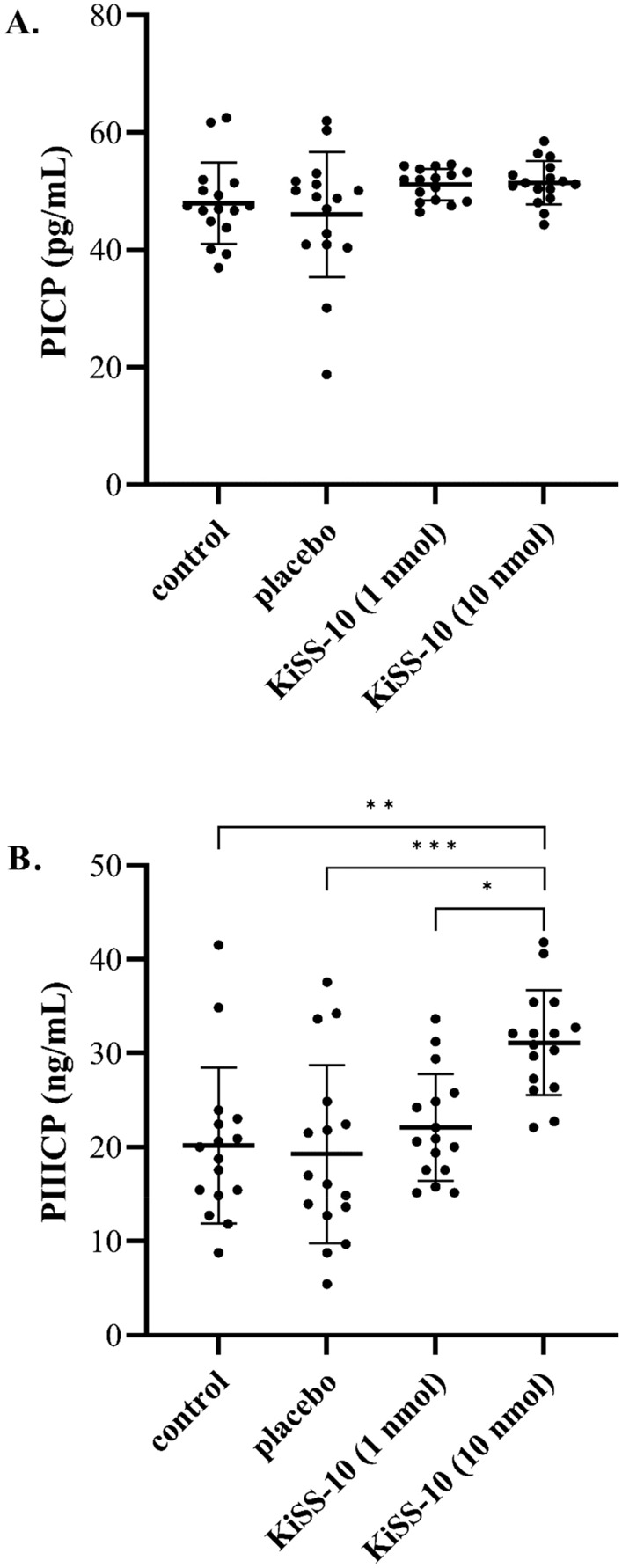
Changes in serum concentration of C-terminal propeptides of procollagen type I (PICP) (**A**) and III (PIIICP) (**B**) in mice: control group; placebo (group receiving injections *aqua pro injectione*); experimental group receiving injections of KiSS-10 (1 nmol/200 µL); experimental group receiving injections of KiSS-10 (10 nmol/200 µL). Experiments were performed in biological replicates (PICP: n = 8, PIIICP: n = 8). Each biological replicate was assayed in duplicate. Error bars represent standard deviation. Data were analysed by the Kruskal–Wallis test (*p < 0.05; **p < 0.01; ***p < 0.001—significant difference between the groups).

The results revealed that treatment of mice with kisspeptin-10 (10 nmol/200 µL) for four weeks markedly elevated the serum concentration of PIIICP (31.12 ± 5.57 ng/mL) compared to the control (20.17 ± 8.31 ng/mL), placebo (19.26 ± 9.48 ng/mL) and mice, which received KiSS-10 at the lower dose of 1 nmol/200 µL (22.08 ± 5.70 ng/mL) (Fig. 8B). However, no significant changes in the serum concentration of PICP were found between all groups in mice in vivo (Fig. 8A).

## Discussion

This study examines the role of kisspeptin-10 in the regulation of collagen metabolism in the heart; in addition, unlike previous studies, it also attempts to determine the mechanism behind the effect of kisspeptin-10 on collagen deposition within the heart, which could be related to cardiac fibrosis. Our in vitro findings indicate that the intracellular collagen content in human cardiac fibroblasts is increased under the influence of KiSS-10 (10^−11^–10^−6^ mol/L). The most stimulatory effect of KiSS-10 on collagen in fibroblasts was observed after exposure to 10^−8^ mol/L of kisspeptin-10. The cells treated with 10^−8^ mol/L of KiSS-10 demonstrated 4.25-fold elevation in collagen content compared to non-treated controls. However, the highest concentration of KiSS-10 (10^−5^ mol/L) did not affect intracellular collagen level compared to the control (Fig. 1A). Moreover, 10^−8^ mol/L of KiSS-10 was found to increase the expression of *Col1A1* and *Col3A1 *in vitro (Fig. 1B,C). By contrast, other studies on Human Aortic Smooth Muscle Cells (HASMCs) have shown that kisspeptin-10 did not significantly alter the protein expression of collagen-1 or collagen-3^21^.

Collagen is synthesized by fibroblasts as pre-procollagen. Cleavage of signal peptide leads to the formation of procollagen molecule. This procollagen is excreted from the cell, releasing the carboxy-terminal or the amino-terminal propeptides, i.e. collagen type I (PICP, PINP) and type III (PIIICP, PIIINP), what results in collagen formation. Thus, these factors can be used as markers of collagen biosynthesis, which are elevated during the overproduction and deposition of collagen in the heart associated with myocardial fibrosis^22^. Our findings indicate that kisspeptin-10 is involved in the regulation of collagen synthesis by cardiac fibroblasts in vitro; the hormone significantly stimulates PICP and PIIICP release from human cardiac fibroblasts compared to controls when applied at concentrations of 10^−6^ and 10^−8^ mol/L (Fig. 2).

Cardiac fibroblasts also produce metalloproteinases (MMPs) and their inhibitors: TIMPs. MMPs are the predominant proteases that are involved in the cleavage of collagen. Thus, it is critical to achieve a balance between the function of MMPs and TIMPs to maintain ECM homeostasis. However, changes in the levels of MMPs and TIMPs are observed under pathological conditions such as cardiac fibrosis. These alterations are dependent on the type, stage and severity of the disease^2^. According to our in vitro studies, kisspeptin-10 inhibits the release of MMPs (MMP-1, -2, -9) compared to the control (Fig. 3), thereby reducing collagen degradation. Lee et al.^23^ also note that kisspeptin-mimicking peptide inhibits the MMP-1 mRNA expression levels, but increases type I procollagen secretion in UV-induced human dermal fibroblasts. The inhibitory influence of kisspeptin on MMPs secretion has also been noted in cancer cells. Ciaramella et al*.*^12^ observed that treatment with KiSS-10 reduces the activity of MMP-2 and MMP-9 in human malignant mesothelioma cells. However, other studies have shown that kisspeptin-10 significantly enhances the activities of MMP-2 and MMP-9 on HASMCs^21^. According to these studies, KiSS-10 also inhibits the activity of MMPs by stimulation of their tissue inhibitors secretion (TIMP-1,-2,-4) (Fig. 4A,B,D). Other reports also indicate that kisspeptin affects MMPs (MMP-1, -2, -3, -7, -9, -10, -14) directly by downregulating their transcription, as well as indirectly by upregulating TIMP transcription (TIMP-1, -3) in trophoblast cells^24^.

Cardiac fibroblasts are also involved in production of growth factors and other signalling molecules that regulate cellular function and protease activity, thereby modulating ECM biosynthesis and breakdown. Fibroblasts secrete, among others, transforming growth factor β1, which is involved in cardiac fibrosis. It is known that TGF-β1 activates fibroblasts and promotes their transition to myofibroblasts, which produce components of the ECM^6^. However, our findings indicate that the addition of KiSS-10 to the human cardiac fibroblasts cultures does not affect secretion of TGF-β1 (Fig. 5A). Therefore, the profibrotic effect of KiSS-10 within the human cardiac fibroblasts is not mediated by TGF-β1 release.

The signalling associated with FAK activity has been reported to participate in fibrogenesis in *inter alia* skin^25^, lungs^26^, liver^27^ and heart^28^. It is known that FAK integrates growth factor and integrin signals to induce myofibroblast differentiation and promote fibrosis formation^29^. Rajshankar et al*.*^30^ demonstrate that FAK activity contributes to tractional collagen remodeling, but also inhibits collagen degradation via metalloproteinase activity in mouse embryonic fibroblasts. Lagares et al*.*^26^ report that FAK expression and activity are upregulated in fibroblasts from lung fibrosis patients. They also noted that pharmacological inhibition of FAK, as well as its siRNA-mediated silencing, ameliorates bleomycin-induced lung fibrosis and reduces collagen deposition in the lungs of mice in vivo. In contrast, Gałdyszyńska et al*.*^31^ reported that inhibition of FAK using 10^−7^ mol/L of FAK kinase inhibitor 14 results in increased collagen content within human cardiac fibroblast culture. Some data suggests that kisspeptin has a stimulatory effect on FAK in some types of cells. Wu et al*.*^32^ noted that kisspeptin regulates the cell motility of endometrial cancer cells through the phosphorylation of FAK and Src-dependent activation of MMP-2. According to Roseweir et al.^33^, kisspeptin-10 also activates FAK in extravillous trophoblast-derived cells and participates in the inhibition of placental trophoblast cell migration. In contrast, it has been demonstrated that kisspeptin agonist decreases the phosphorylation of FAK, while the antagonist increases it in human decidual stromal cells^34^. Our present in vitro experiment found that 10^−8^ mol/L KiSS-10 stimulates phosphorylation of FAK at Tyr397 in human cardiac fibroblasts compared to controls (Fig. 5B). Moreover, since kisspeptin-10 increases the level of phospho-FAK in cardiac fibroblasts, the study examined whether this signaling pathway mediates the effect of KiSS-10 on collagen deposition. It was found that the cells treated with both KiSS-10 (10^−8^ mol/L) and FAKi (10^−6^ mol/L) demonstrated a significant reduction intracellular collagen content compared to the culture with KiSS-10 alone (Fig. 5C). Thus, FAK inhibitor autophosphorylation inhibition of FAK at Y397^35, 36^ abolishes the stimulatory effect of KiSS-10 on collagen level in human cardiac fibroblasts. These observations clearly confirm that the profibrotic influence of KiSS-10 on collagen content is associated with FAK activity. However, kisspeptin activates other signaling pathways and further studies are needed to determine whether they could be also involved in collagen metabolism within the heart.

In vivo studies with a mouse model confirmed that kisspeptin-10 has an influence on collagen deposition in the myocardium. A significant increase in collagen content was noted in the hearts of mice from the experimental group, which received an injection of kisspeptin-10 (10 nmol/200 µL), compared to the control and placebo (Fig. 6). The obtained results also indicate that a significant increase in collagen content in the hearts of mice is associated with an elevation of the collagen biosynthesis marker PIIICP. Treatment of mice with 10 nmol/200 µL kisspeptin-10 for 4 weeks significantly increased the serum concentration of PIIICP compared to the other groups (Fig. 8B). However, in addition, a significant decrease in the expression of *Col1A1* and *Col3A1* was observed in the experimental groups, i.e. which received kisspeptin-10, compared to the control group and placebo (Fig. 7). The obtained data suggest that kisspeptin-10 could promote collagen accumulation in the heart in vivo due to the intensification of collagen biosynthesis at post-transcriptional levels. It is known that co- and posttranslational modifications are required for formation of functional collagen. The crucial role plays enzymatic modification of proline and lysine residues under the influence prolyl hydroxylase and lysyl hydroxylase. Also, there are other enzymes that are involved in collagen biosynthesis and formation of covalent intermolecular cross-linking, that is critical for stabilization of collagen. Lysyl oxidase is a copper-dependent monoamine oxidase that mediates collagen cross-linking in the extracellular matrix. However, it is difficult to confront the results of this study with data from the literature because no other reports on the effect of kisspeptin on activity of enzymes that are involved in collagen synthesis have been published so far^37, 38^. This stimulatory effect of kisspeptin on collagen deposition and development of cardiac fibrosis was also noted in rats. Zhang et al.^8^ reported that treatment with kisspeptin-10 for seven days leads to myocardial changes and cardiomyocyte damage, including an increase in the numbers of collagen fibers in the cardiac muscle and a certain degree of fibrosis, as well as myocardial contractions or mitochondrial cristae fracture. By contrast, studies on lungs have shown that kisspeptin-13 inhibits bleomycin (BLM)-induced pulmonary fibrosis in mice through decreased collagen/α-smooth muscle actin deposition and suppression of the inflammatory response^39^. Moreover, studies indicate that hepatic KiSS1R deficiency promotes liver fibrosis biomarkers in a high-fat diet (HFD)-induced mouse model. On the contrary, activation of hepatic KISS1R plays a protective role against steatosis and reduces fibrosis of the liver in a diet-induced mouse model of non-alcoholic liver disease^40^.

## Conclusion

To conclude, KiSS-10 appears to be involved in the regulation of collagen metabolism in the heart. The augmentation of the collagen deposition observed under the influence of kisspeptin-10 is dependent on an elevation of protein synthesis, inhibition of matrix metalloproteinase activity (increase of TIMPs release) or decrease of MMPs concentration. This effect of KiSS-10 is related to its direct action on human cardiac fibroblasts. In addition, this profibrotic activity is mediated by the activity of FAK. These findings indicate that kisspeptin-10 could be involved in pathological cardiac remodeling, and that modulating KiSS-10 concentrations in the body may serve as a new therapeutic approach that could regulate collagen metabolism in the heart and stabilize the connective tissue matrix.

## Data Availability

The data will be made available from the corresponding author on reasonable request.
